# CNS Involvement of Multiple Myeloma—A Case Series and Narrative Literature Review

**DOI:** 10.3390/jcm15103927

**Published:** 2026-05-20

**Authors:** Andreea Andrunache, Mihai Emanuel Himcinschi, Sinziana Barbu, Larisa Emilia Zidaru, Didona Alexa, Monica Popescu, Sara Mihaela Apuscaroaie, Delia Codruta Popa, Iulia Ursuleac, Daniel Coriu, Sorina-Nicoleta Badelita

**Affiliations:** 1 Haematology and Bone Marrow Transplant Centre, Clinical Institute Fundeni, 022328 Bucharest, Romania; 2 Haematology Department, University of Medicine and Pharmacy Carol Davila, 020021 Bucharest, Romania; 3 Department of Biochemistry, Victor Babes University of Medicine and Pharmacy, 300041 Timisoara, Romania

**Keywords:** multiple myeloma, extramedullary disease, CNS involvement

## Abstract

**Background**: Central nervous system (CNS) involvement in multiple myeloma (MM) represents an extramedullary manifestation of the disease, which is often really challenging for clinicians, as the neurological symptoms could easily overlap with those related to hypercalcemia, uremia, high viscosity of the blood, or treatment-related neuropathy. **Objectives**: this retrospective study was conducted at Fundeni Clinical Institute in Bucharest, aiming to identify and systematically analyze a series of clinical cases diagnosed with extramedullary disease. **Methods**: We have identified 6 out of 583 patients with CNS involvement in our centre between 2019 and 2025. The diagnosis of meningeal myelomatosis was established through cerebrospinal fluid analysis, whereas CNS plasmacytomas were confirmed by CT-guided biopsy followed by immunohistochemistry evaluation. **Results**: All cases of CNS involvement occurred at relapse, with intervals from initial MM diagnosis to CNS involvement ranging from 9 months to 10 years. CNS-MM was linked to particular features, such as high-risk cytogenetics (four out of six patients), elevated lactate dehydrogenase, and the presence of extramedullary disease, highlighting its association with aggressive disease behaviour. **Discussions**: Although CNS-MM is correlated with poor prognosis, prolonged survival in one of our patients resulted from multimodal treatment, which included craniospinal radiotherapy, DPd systemic treatment, and intrathecal therapy (over 39 months). This aggressive approach effectively controlled both systemic disease and high-risk CNS involvement. Immunoglobulin isotype switching is a rare form of clonal evolution in MM, illustrated by the same patient whose disease evolved from IgA kappa at diagnosis to IgA lambda at CNS relapse, showing clonal heterogeneity and providing clinical evidence of clonal evolution. **Conclusions**: CNS involvement in MM usually occurs in a relapsed/refractory setting in patients with advanced, high-risk disease, and it is usually associated with extramedullary disease. Despite using multimodal therapies, outcomes remain poor, highlighting the need for novel and tailored agents.

## 1. Introduction

Multiple myeloma (MM) is one of the most common haematological malignancies characterised by clonal proliferation of abnormal plasma cells, usually driven by genetic abnormalities [1]. Although the new therapies have significantly improved survival, this disease remains incurable as patients ultimately develop resistance to therapy [2].

Extramedullary disease (EMD) represents an aggressive form of multiple myeloma characterised by plasma cell proliferation outside the bone marrow, in tissues such as the liver or central nervous system. EMD is more common in relapsed patients and has an unfavourable prognosis with limited response to treatment [3,4].

Central nervous system MM (CNS-MM) can occur both at the onset of myeloma or, more commonly, at the onset of relapsed/refractory disease. In a multicentre study, Landry et al. [5] observed that most patients developed CNS involvement after multiple lines of therapy, with a median interval of 19 months from initial diagnosis of MM to the onset of neurological manifestations. Survival of patients with CNS-MM at initial presentation is longer than that of those who develop it after multiple lines, but remains significantly shorter compared with patients without extramedullary involvement. Indicative symptoms include persistent headache that is usually refractory to common analgesics, emetic syndrome, palsy of cranial nerves or pain due to spinal root involvement, cognitive dysfunction, or seizures. Diagnosis and monitoring of CNS multiple myeloma requires a multidisciplinary strategy and coordinated efforts to improve the patient’s survival and quality of life.

Retrospective studies, such as the multicentric study of 172 patients [6] and a nationwide study from Japan of 77 patients [7], report a consistent incidence of approximately 1% among all patients with MM. Similarly, a real-world analysis from Greece, which evaluated 4352 patients, identified CNS-MM in 1.2% of them [8].

The prognosis of CNS-MM is significantly worse when correlated with the presence of major risk factors, such as adverse cytogenetic features (17p, 1q+ deletion), systemic extramedullary disease, elevated LDH levels, and the occurrence of plasma cell leukaemia [8,9].

This retrospective study was conducted at Fundeni Clinical Institute in Bucharest, aiming to identify and systematically analyse a series of clinical cases diagnosed with extramedullary disease. Among the 583 patients diagnosed with MM at our Institute between 2019 and 2025, we identified 6 with CNS involvement.

The aim of our study was to characterise a small cohort of patients diagnosed with secondary CNS involvement in multiple myeloma. In the absence of dedicated treatment protocols, this study contributes to the experience of our department in managing this rare condition, which requires individualised therapeutic strategies to improve survival. Standard practice dictates that every patient should receive a tailored therapeutic approach, and even though survival is generally poor, we aim to recognise the situations where survival can be increased.

## 2. Materials and Methods

The narrative review segment of the manuscript is based on a non-systematic literature search. The PubMed database was consulted to identify relevant English-language articles using the following keywords: “CNS involvement,” “multiple myeloma,” and “extramedullary disease.” The search covered the period from 2002 to 2026. A total of 32 references were initially identified. After manual selection based on relevance, methodological quality, and expert judgment, 25 references were retained for inclusion in this review. The selection of references was based on the authors’ expert judgment to provide a comprehensive overview of the topic.

For the case report segment, we conducted a retrospective, observational, single-centre analysis of data from the National Multiple Myeloma Registry. The study was conducted in a tertiary haematology centre in Romania. The analysis included adult patients (age over 18 years) diagnosed with multiple myeloma between January 2019 and January 2025, with the diagnosis confirmed according to standard international criteria.

This study was conducted within the Department of Haematology of the Fundeni Clinical Institute, Bucharest, in accordance with the Declaration of Helsinki and its amendments. All patient data were anonymised before statistical analysis and interpretation of the results. At the time of every hospital admission or follow-up visit, all patients provided written informed consent agreeing to the inclusion of their data in the institutional electronic registry for future research purposes. The national Multiple Myeloma Registry operates under ongoing institutional approval for clinical data management. The protocol of this study was approved by the hospital ethics committee (12786/13.03.2026), giving us the permission to interrogate the database registry. No data was analysed prior to this approval or without patient consent.

**Figure 1:**
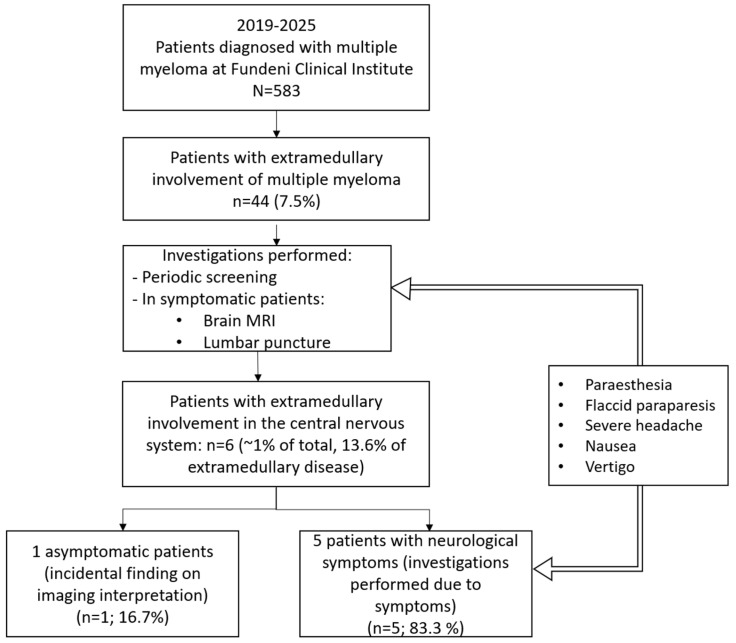
Flowchart of patient selection for CNS involvement analysis in multiple myeloma at the Fundeni Clinical Institute (2019–2025). Of 583 patients diagnosed with multiple myeloma, 44 had extramedullary disease and 6 presented CNS involvement, identified through periodic screening or symptom-driven investigations, including brain MRI and lumbar puncture. Five patients were symptomatic, while one case was detected incidentally.

Of these, six patients (1.03%) met the diagnostic criteria for CNS involvement, and all six are reported in this case series (Figure 1 below shows the flowchart of patient selection for CNS involvement). The small sample size (*n* = 6) does not allow for inferential statistical analysis or meaningful subgroup comparisons. Our findings are therefore descriptive and hypothesis-generating rather than definitive. Demographic and clinical data were extracted from the electronic medical records. Treatment regimens were analysed, and the response was evaluated according to the International Myeloma Working Group criteria. The diagnosis was based on the presence of neurological symptoms in patients with previously diagnosed multiple myeloma [7,10,11].

Our patients who indicate a high suspicion for CNS involvement are recommended for further evaluation using brain and spinal cord imaging (MRI and CT) to detect leptomeningeal and cerebral parenchymal involvement. Further diagnostic evaluation included performing a diagnostic lumbar puncture and extensive testing of the CSF, which includes electrophoresis and determination of CSF free light chains. Laboratory assessment comprises cultures, measuring the levels of glucose and proteins in the CSF. Flow cytometry tests can be performed as well if necessary [12,13].

Cerebrospinal fluid was centrifuged, smeared, fixed, and Giemsa-stained for cytological examination. Under the microscope, pathological cells—especially plasma cells with variable morphology—were identified and quantified, alongside other elements like mesothelial cells, macrophages, and peripheral blood cells.

## 3. Results

In accordance with the study design described above, in a cohort of 583 patients with multiple myeloma (MM), CNS involvement was a rare complication (1.03%, *n* = 6). The median age at diagnosis of MM was 58.5 years, and at diagnosis of CNS involvement was 61.5 years. All cases occurred at the time of relapse, with a median interval of 3.75 years (9 months–10 years). Bone marrow infiltration at the time of relapse was variable (median 35.5%) and did not correlate with CSF protein levels (ρ = 0.09), suggesting that the CNS may act as a sanctuary in which systemic therapy has limited action. The clinical course of the two most representative cases will be presented below in detail. The patients’ demographic data, clinical characteristics, treatment courses, and therapeutic approaches are summarised in Table 1, Table 2 and Table 3.

Patient 2

The second patient was a 63-year-old patient, known with a diagnosis of stage I ISS IgA kappa multiple myeloma since 2011. His evolution was favourable after a series of Melphalan–Cyclophosphamide–Prednisone courses. Initial laboratory assessment included a bone marrow biopsy that revealed 90% plasma cell infiltration, while serum protein electrophoresis yielded a monoclonal spike of 2.17 g/dL, elevated levels of IgA, and free kappa chains.

**Figure 2:**
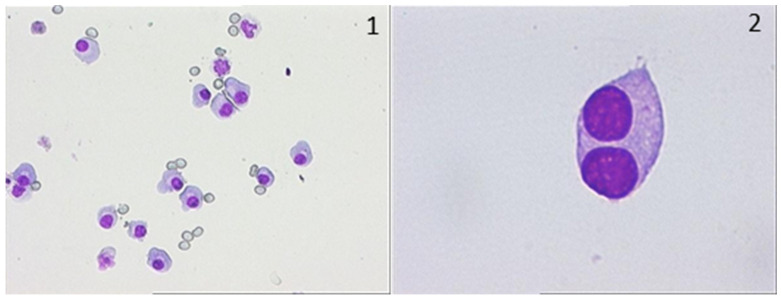
May–Grunwald Giemsa colouration; optical captures of CSF smear; multiple plasmacyte infiltrate of CSF of PAT_2, 40× magnification (**1-left**); binucleated plasmacyte infiltrated in CSF of PAT_2, 100× ob (**2-right**).

In April 2020, the patient presented with flaccid paraparesis and was admitted to the neurology department. MRI revealed contrast enhancement in the cauda equina nerve roots along with leptomeningeal gadolinium uptake of the conus medullaris. Lumbar puncture revealed significantly elevated proteins and lymphocytosis in the cerebrospinal fluid. Suspecting herpes simplex encephalitis, antiviral treatment for HSV was initiated, which resulted in minimal clinical improvement. For the next five months, the patient remained confined to a wheelchair and subsequently developed urinary incontinence, requiring bladder catheterisation. In March 2021, he presented with increasing pain and deterioration in his general condition, raising the suspicion of central nervous system involvement. Tests revealed slightly elevated LDH levels, with no other signs of active disease. EMG did not confirm polyneuropathy. MRI of the brain without acute changes and analysis of the cerebrospinal fluid showed increased proteinuria and the presence of lymphoplasmacytic cells and binuclear plasma cells. A smear was made using the same CSF sediment, revealing lymphoplasmacytic cells, frequent binucleated plasma cells, and relatively frequent monocytes, as shown below in Figure 2.

**Figure 3:**
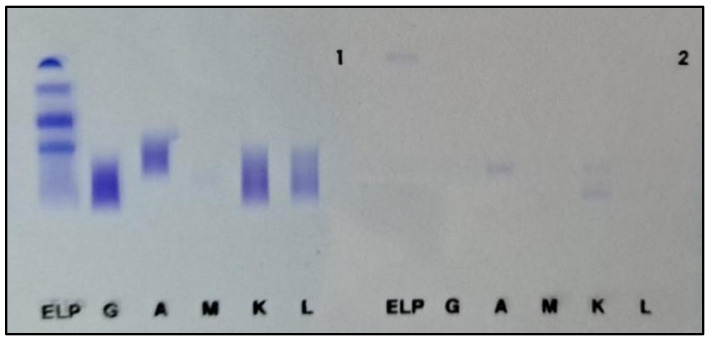
Cerebral spinal fluid immunofixation of Patient 2, showing high concentration for alpha heavy chains and kappa light chains (**1-left**); serum fluid immunofixation of Patient 2 showing negligeable fixation of alpha heavy chains and kappa light chains (**2-right**).

While CSF immunofixation was positive for heavy alpha chains and kappa light chains, serum immunofixation remained negative (Figure 3). Immunophenotyping of the CSF revealed myelomatous plasma cells, positive for CD138, CD38 and intracellular expression of kappa chains and negative for CD19, CD20, and CD45.

The patient remained ISS Stage I, but with signs of active tumour burden. In accordance with international guidelines, systemic treatment with DRd was initiated, accompanied by intrathecal chemotherapy administration (five lumbar punctures—MTX, ARA-C, DXM). The patient developed severe and prolonged pancytopenia, which led to the onset of pneumonia and subsequently acute respiratory failure. The patient experienced multiple urinary tract infections as well, before presenting in our clinic, most likely due to disease-related immunosuppression. Given the progressive deterioration, the patient was admitted to the intensive care unit, where he passed away.

Patient 3

The third patient, a 64-year-old male with ankylosing spondylitis, presented with progressive lower back pain irradiating to the lower limbs. Imaging revealed a destructive vertebral mass at T12-L1 with epidural extension and compression of the dural sac, diagnosed as plasmacytoma. Initial hematologic workup from our clinic found normal LDH, elevated b2m and IgA levels. Immunofixation was positive for kappa light chains, and bone marrow aspiration counted 28–30% small plasma cells. The patient was negative for t(4;14), t(14;16) and del17p on the FISH exam.

**Figure 4:**
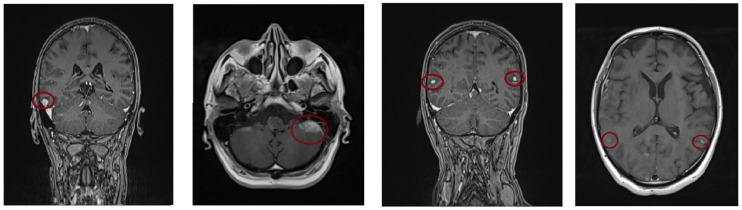
Brain MRI: multiple nodular meningeal lesions with intense contrast enhancement, located bilaterally in supratentorial and infratentorial regions, including the left cerebellar area. Imaging findings are suggestive of secondary meningeal involvement in the setting of multiple myeloma.

The diagnosis of IgA kappa stage II multiple myeloma (ISS) was made in November 2018. The patient underwent induction therapy with CyBorD, followed by autologous hematopoietic stem cell transplantation, achieving a complete response, and subsequent radiotherapy for bone lesions. In September 2020, a biological evaluation showed an increase in kappa free light chains to 711 mg/L. Bone marrow examination revealed 39% small plasma cells, and FISH analysis was negative for t(4;14), t(14;16), t(11;14), and del17p but positive for deletion 1p in 52% of cells. Due to disease progression, the patient received nine cycles of KRD in a clinical trial. Carfilzomib treatment was discontinued after a non-ST-segment elevation myocardial infarction (NSTEMI) due to drug toxicity. Three further cycles of RD were then administered, after which treatment was discontinued until March 2022, maintaining very good VGPR. In March 2022, the patient presented with severe occipital headache, diplopia, and blurred vision. Brain MRI revealed multiple nodular meningeal lesions with a tumour-like appearance and intense contrast enhancement. Additional lesions were noted in the right capsulo-lenticular area, the largest measuring approximately 27/14 mm along the anterior surface of the left cerebellar lobe (Figure 4).

**Figure 5:**
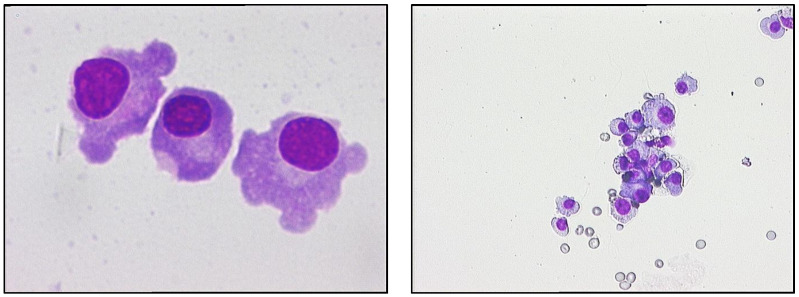
May–Grunwald Giemsa colouration; optical captures of CSF smear; multiple plasmacyte infiltrate of CSF of PAT_3, 100× magnification (**left**); plasmacyte infiltrated in CSF of PAT_3, 40× ob (**right**).

CSF laboratory assessment showed a normal glucose level, while the protein concentration was markedly elevated. CSF cultures remained negative, ruling out acute bacterial infection. Cytological examination of CSF (Figure 5) showed a highly cellular smear predominantly formed of plasma cells, with only very rare granulocytes observed.

Flow cytometric analysis revealed approximately 99% of the total cellular population exhibited the following immunophenotype: weak CD45 expression, strong CD38 expression, and negativity for CD19, CD20, CD16, CD5, and CD3, with positivity for CD56—consistent with a profile characteristic of malignant plasma cells.

**Figure 6:**
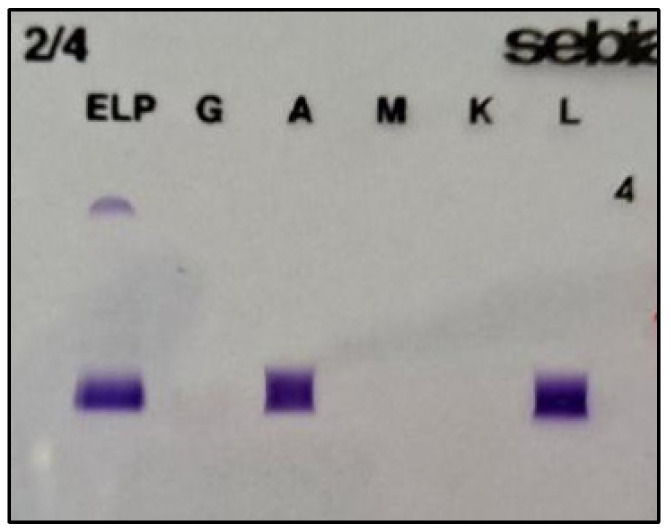
Cerebral spinal fluid immunofixation of patient 3, showing high concentration for alpha heavy chains and lambda light chains.

On the other hand, CSF immunofixation was positive for lambda free chains. Blood workup showed normal levels of LDH, b2m, and immunoglobulins, but an elevated free lambda chains level of 131 mg/L, while serum immunofixation was positive for lambda chains. A notable finding in this case is the isotype switch from IgA kappa at initial diagnosis to IgA lambda at the time of CNS relapse, reflecting a potential shift in the dominant malignant clone. Results of the immunofixation are presented in Figure 6.

The patient was diagnosed with myelomatous meningitis, and he received a total of 39 courses of DPd, along with 8 intrathecal administrations with MTX, DXM and ARA-C. Additionally, craniospinal radiotherapy was performed, delivering a total dose of 31.5 Gy in 21 fractions. Last follow-up lumbar puncture showed no myeloma cells in the cerebrospinal fluid, and he currently continues to maintain the response to the DPd regimen.

## 4. Discussion

Central nervous system involvement of multiple myeloma remains a rare occurrence [6,10,11,14,15], with a literature incidence of 0.5–1.2% in documented cases of MM [6,12,16,17]. In our cohort of 583 patients, only six patients (1.03%) were diagnosed with CNS multiple myeloma between 2019 and 2025. The median age at diagnosis of multiple myeloma was 58.5 years (range: 42–62 years), with equal sex distribution (male:female ratio is 1:1). Furthermore, the median age at diagnosis of CNS involvement described in the literature ranges from 50 to 63 years across different studies [6,7,8,16], which is consistent with the median age in our cohort of 61.5 years (range: 47–70). All of our cases emerged at relapse, with the interval from initial diagnosis of MM to CNS relapse ranging from 9 months to 10 years (median time to CNS relapse is 3.75 years). This observation is in line with the previous reports, which mention a remarkably wide interval as well, from 0 months to up to 15 years [7,8,16].

Bone marrow infiltration at relapse was highly variable: some patients showed progression of the disease, while others presented no involvement. Median plasma cell infiltration was approximately 35.5% (range: 10–100%). In this small cohort, no apparent correlation was observed between CSF protein levels and bone marrow plasma cell infiltration at CNS relapse (Spearman ρ = 0.09), indicating that CSF biochemical results may not reflect systemic tumour burden. This aspect may be clinically relevant because systemic treatment strategies may not target some sanctuary sites, such as the CNS.

CNS involvement in MM presents with heterogeneous non-specific symptoms, such as persistent headache resistant to common analgesics, diplopia, emetic syndrome, which could delay diagnosis [7,11]. In our cohort, symptoms ranged from focal neurological deficits to no symptoms. PAT_2 presented with urinary incontinence, severe radicular pain, while PAT_3 had severe occipital headache, horizontal diplopia and blurred vision. In contrast, PAT_5 presented acute vertiginous syndrome, accompanied by nausea and headache. PAT_4 presented an insidious onset of the disease, which developed to extreme lethargy, while PAT_6 was entirely asymptomatic, and the CNS involvement was discovered incidentally. Despite its rarity and non-specific symptomatology, this entity should remain in the differential diagnosis when clinical suspicion arises.

Literature indicates that MRI is the method of choice, with high sensitivity (90%) for detecting leptomeningeal and parenchymal involvement [6,8,18]. CT scan, which is less sensitive (≈81%), may be used when MRI is not available [6,17]. In contrast to leptomeningeal involvement, Manzar et al. [19] reports that localised cerebral involvement is associated with better overall survival and a higher rate of durable complete remission. In our cohort, four out of six patients presented with brain parenchymal involvement via intracranial masses detected through brain imaging, while the other two presented with leptomeningeal involvement. In both situations, imaging proved its usefulness.

Furthermore, for our symptomatic patients (PAT_1, PAT_2, PAT_3, PAT_5), cerebral MRI with gadolinium contrast revealed different patterns of the disease: nodular or plaque-like leptomeningeal enhancement (PAT_3), solitary or multiple intraparenchymal enhancing masses (PAT_1, PAT_5), or nerve root enhancement (PAT_2), while spinal MRI revealed epidural extension (PAT_3, PAT_4). On the other hand, CT imaging, although less sensitive for the detection of CNS involvement [20], provided enough information to guide further diagnostic evaluation in PAT_6, in which a routine WBLD-CT scan incidentally revealed an intracranial tumoral lesion. PET-CT scan was used for PAT_4, PAT_5 and identified metabolically active extramedullary and intracranial lesions. As lytic skull lesions that extend into the epidural space can cause significant intracranial pressure even when neurological symptoms are absent, we acknowledge the importance of active imaging surveillance in high-risk patients.

Neuroimaging findings should be integrated with cerebrospinal fluid analysis, and, where feasible, histopathological examination of the cerebral lesions to confirm the diagnosis. CSF cytology and immunophenotyping were used to identify the plasma cells for all cases. The biochemical pattern was characterised by elevated protein levels, most pronounced in PAT_2, PAT_3, and PAT_5; normal or mildly elevated glucose concentrations were observed. Cerebrospinal fluid immunofixation showed evidence of clonal restriction, specifically IgA kappa in PAT_2 and lambda light-chain restriction in PAT_3. In contrast, stereotactic biopsy of intracerebral lesions was performed for PAT_1, and it revealed a diffuse myelomatous proliferation, confirming leptomeningeal involvement that could not be established by cerebrospinal fluid analysis alone.

Patient 3 shows a particular pattern of the disease, manifested by a shift in the dominant immunoglobulin isotype. The patient’s initial disease was characterised as IgA kappa multiple myeloma, confirmed by serum immunofixation. At the onset of myelomatous meningitis, the laboratory assessment revealed serological and CSF discordance. Systemic evaluation showed a low IgA level with high lambda serum chains. This biochemical shift was confirmed in CSF, where immunophenotyping was positive for lambda light chains and negative for kappa. The complex clonal evolution and heterogeneity of multiple myeloma, immunoglobulin isotype switching (from IgA kappa to IgA lambda—the case of PAT_3) is a rare clinical manifestation [21]. It can occur either as a result of clonal evolution or as a benign phenomenon associated with post-therapy immune reconstitution, according to Liang et al., 2022; Li et al., 2023 [15,22].

The literature consensus is that the efficiency of treatment for this group of patients with extramedullary disease is directly correlated with their capacity to penetrate into the cerebrospinal fluid. As noted by several authors [5,11,16], bortezomib, carfilzomib, and lenalidomide achieve only low therapeutic concentrations in the CSF. On the other hand, the category of drugs that have good penetration includes pomalidomide and Selinexor [23]. Our approach to CNS involvement used a multimodal strategy combining CNS-directed therapy and novel systemic immunotherapies, but the outcomes remained variable, highlighting the ongoing therapeutic challenge. We observed that CNS involvement sometimes developed even when systemic disease was well controlled. These findings could indicate that the CNS may act as a sanctuary site where therapies are not considered as efficient, allowing relapse to occur, but further research is needed for confirmation.

Intrathecal therapy with MTX, ARA-C and DXM is frequently used in the management of CNS-MM, especially in leptomeningeal forms, being administered in 32–43% of patients [6,7,8]. The use of liposomal ARA-C resulted in a positive response in 43% of patients, with a median duration of response of 2.5 months and manageable toxicity [24]. In Jurczyszyn’s analysis [6], intrathecal administrations were part of a multimodal treatment that resulted in a median survival of 7 months, which is elevated when compared with patients who did not receive intrathecal therapy (2 months).

For local disease control, we used intrathecal MTX, DXM, and ARA-C for confirmed leptomeningeal involvement (PAT_2, PAT_3) and craniospinal or stereotactic radiotherapy for symptomatic mass lesions (PAT_1, PAT_3, PAT_5). An important observation was the discordance between deep systemic response and CNS progression. PAT_2 developed CNS relapse after a 10-year treatment-free interval, without systemic progression. At the same time, PAT_5 achieved a very good partial sustained systemic response with anti-BCMA bispecific therapy but later developed brain masses, while PAT_3 had durable CNS remission after early combined local and systemic treatment. Although systemic responses were observed in these cases, this raises the question of whether novel immunotherapies may have limited capacity to control CNS disease, a possibility that requires further study.

Another therapeutic approach used in patients with central nervous system involvement is radiotherapy, which has been administered in various studies to 36% to 78% of patients with brain involvement [6,7,12]. In a multivariate analysis of the Japanese study, RT was associated with significantly longer survival (HR 0.33; *p* < 0.001) [7]. The MD Anderson series on 45 patients reported complete responses in 66.7% of evaluable cases post-RT, with a median survival of 7.3 months in complete responders and survivals of over 11 months in six patients, especially in focal brain forms [19]. Published studies have reported that radiotherapy is associated with significantly longer survival in CNS-MM [7,17,19]. In our series, three patients underwent CNS-directed radiotherapy. For example, the third patient received a total dose of 31.5 Gy in 21 fractions, contributing to a durable CNS remission of over 39 months. This is consistent with literature reports, and it highlights the benefit of RT in CNS involvement, although our small sample does not allow any definitive conclusion regarding survival.

Systemic therapy represents an important component in the management of central nervous system involvement. In published series, systemic treatment has been used in 76% to 97% of patients [7]. Immunomodulatory agents are frequently used due to their ability to penetrate the cerebrospinal fluid. Pomalidomide demonstrates the highest CSF penetration, approximately 40% of the corresponding plasma concentration, followed by lenalidomide and thalidomide (11–49% and 30–60%, respectively) [5]. Proteasome inhibitors have limited CNS penetration; for example, bortezomib reaches a concentration of only 5–7% in the plasma [5,23]. Monoclonal antibodies such as daratumumab can be detected in the CSF, although typically not at therapeutically optimal concentrations. Nevertheless, Varga et al. report complete responses with daratumumab in combination with triple intrathecal therapy [11].

In our cohort, PAT_2 and PAT_3, who presented leptomeningeal involvement, received systemic therapy with daratumumab-based regimens (DRd and DPd, respectively) combined with intrathecal chemotherapy. PAT_3 achieved a prolonged CNS remission (>39 months), while PAT_2 died shortly after treatment initiation due to an infectious disease. These observations align with literature reports suggesting that daratumumab may help control CNS involvement in some cases [11]. Similarly, one patient (PAT_5) developed new brain masses while on BCMA-targeted bispecific antibody therapy, despite having sustained systemic very good partial response. This raises the hypothesis that immunotherapies may not penetrate into the CNS sanctuary, a concern already stated in the literature [18,25].

Study Limitations: The results should be interpreted in the context of the small number of patients (*n* = 6), which reflects the rarity of CNS involvement in multiple myeloma (~1%). We included all consecutively identified patients as they were the only ones available. We did not have full access to all data for patients partially followed for a limited time in other haematology departments, which may introduce selection bias. There is no characteristic protocol for this rare disease, and the disease is characterised by highly variable symptoms and heterogeneous therapeutic responses. Patients received various systemic treatment regimens (CyBorD, VRD, KRd, DRd, Dara-Kd, bispecific antibodies, CAR-T), and various CNS-targeted therapies, reflecting the lack of standardised protocols.

## 5. Conclusions

The central nervous system’s involvement in multiple myeloma, including myelomatous meningitis and intracranial plasmacytomas, was observed, in our cohort, only in a relapse/refractory context, typically after many lines of treatment, pointing towards its association with an advanced disease biology and an elevated risk factor. The clinical presentation was heterogeneous and often non-specific, including paraesthesia, flaccid paraparesis with sphincter dysfunction, headache with diplopia and vertigo, which may delay the identification of the CNS relapse.

In our series, patients with extramedullary disease had a higher frequency of concomitant or subsequent CNS involvement, supporting further investigation of CNS sites for disease progression.

The treatment required multimodal methods directed at the CNS, including intrathecal chemotherapy, systemic therapy and radiotherapy. Despite these interventions, the outcomes remain reserved in most cases, pointing towards the need for therapies with improved efficiency at the CNS level and for prospective studies which should better help identify the optimal treatment.

## Figures and Tables

**Table 1:** Patients’ characteristics at MM diagnosis.

Variable	PAT_1	PAT_2	PAT_3	PAT_4	PAT_5	PAT_6
Age (years)	43	63	64	54	42	69
Sex	F	M	M	F	F	M
Albumin (g/dL)	4.99	3.8	3.6	3.2	4.1	3.3
M-component (g/dL)	absent	2.17	1	0.4	absent	2.4
Bone marrow infiltration (%)	12%	90%	28–30%	20%	90%	20%
FISH anomalies	Not performed Dry tap	Not performed	Neg: t(4;14), t(14;16), del17p	Pos: del17p (10%), 1q amp	Neg: t(14;16), del17p, t(4;14)	Neg: del17p, 1q abn, t(4;14)
Type of MM	Lambda micromolecular	IgA kappa	IgA kappa	IgD lambda	Kappa micromolecular	IgA kappa
ISS	III	I	II	II	II	III
R-ISS	III	NA	II	II	II	II
B2M (mg/L)	5.9	2.1	4.2	2.3	3.6	>16
LDH (u/L)	413 (High)	182 (normal)	228 (Normal)	292 (High)	222 (Normal)	74 (Low)

**Table 2:** Patients’ characteristics at MM-CNS diagnosis.

Variable	PAT_1	PAT_2	PAT_3	PAT_4	PAT_5	PAT_6
Age at CNS relapse (years)	47	73	67	56	48	70
Dx to CNS relapse (years)	4	10	3.5	2	5.8	1
Key neurological symptoms	Paraesthesia on the right side of the face	Flaccid paraparesis, urinary incontinence	Severe occipital headache, horizontal diplopia, blurred vision	Extreme lethargy	Acute vertigo, nausea, severe headache	No clinical findings
Imaging	-Cerebral CT/MRI: Hyperdense intracranial lesion in right frontal region	-MRI: Contrast enhancement of lumbar cauda equina & leptomeningeal uptake of conus medullaris	-MRI: Multiple nodular/plaque-like leptomeningeal lesions in supra- and infratentorial regions	-MRI: Intracranial dissemination with masses in both cerebral hemispheres	-Cerebral CT: Multiple intra-axial nodular lesions with contrast enhancement and oedema	-Incidental finding on the WBLD CT scan
BM infiltration (%)	20%	<10%	39%	32%	98–100%	90%
FISH	NA	Negative	*1p del* (52%)	*del17p* (40%), *1q amp* (24%) 1 year before	*1q+*	*1q amp* (72%), *t(4;14)* (32%), anormal pattern for *t(14;16)*; *t(11;14)*; *t(14;20)*
Serum B2M (mg/L)	2.5	2.4	1.6	1.4	2	7.2
Monoclonal spike (g/dL)	absent	absent	absent	absent	absent	0.7
Serum albumin (g/dL)	3.8	3.9	4.5	4	4.8	4
LDH (u/L)	157 (Normal)	468 (High)	273 (Normal)	330 (Normal)	686 (High)	149 (Normal)
CSF protein mg/L	234 (N)	1184 (High)	4615 (High)	105 (N)	784 (High)	290 (N)
CSF glucose mg/dL	62	66.3	61	73	78	53
CSF cytology/Flow	Negative	Positive	Positive	Negative	Negative	Negative
CSF immunofixation	Negative	IgA kappa	Negative	Negative	Kappa	Kappa

**Table 3:** Overall timeline of therapeutic approach.

Patient	PAT_1	PAT_2	PAT_3	PAT_4	PAT_5	PAT_6
Initial Therapy	CyBorD	Mel + CFA + DXM	CyBorD + ASCT	VRD + ASCT	CyBorD + ASCT + Lenalidomide	DRd
Key Subsequent Therapies	PAD → KRd → DV-PACE	DRd (started at CNS relapse)	KRd → DPd	RAD → PACE-Pd → Dara-Kd → CAR-T → BCMA bsAb	Dara-Kd → PomVD-PACE → GPRC5D bsAb → BCMA bsAb	-
CNS Relapse Occurred During	During follow-up after KRd	CNS relapse was the event that prompted the start of first-line therapy	During follow-up after KRd	During/after 2 cycles of Dara-Kd	After 16 cycles of BCMA bsAb	DRd
CNS-Directed Therapy	Stereotactic procedure on the brain lesion	IT	IT + RT	IT	RT	-

ASCT—autologous stem cell transplantation; BCMA bsAb—BCMA bispecific antibody; CAR-T—chimeric antigen receptor T-cell therapy; CFA—cyclophosphamide; CyBorD—cyclophosphamide, bortezomib, dexamethasone; Dara-Kd—daratumumab, carfilzomib, dexamethasone; DPd—daratumumab, pomalidomide, dexamethasone; DRd—daratumumab, lenalidomide, dexamethasone; DV-PACE—dexamethasone, bortezomib, cisplatin, doxorubicin, etoposide, cyclophosphamide; DXM—dexamethasone; GPRC5D bsAb—GPRC5D bispecific antibody; IT—intrathecal therapy; KRd—carfilzomib, lenalidomide, dexamethasone; Mel—melphalan; PAD—bortezomib, doxorubicin, dexamethasone; PACE-Pd—PACE (cisplatin, doxorubicin, cyclophosphamide, etoposide) plus pomalidomide and dexamethasone; PomVD-PACE—pomalidomide, bortezomib, dexamethasone plus PACE; RAD—lenalidomide, doxorubicin, dexamethasone; RT—radiotherapy; VRD—bortezomib, lenalidomide, dexamethasone.

## Data Availability

All data can be made available by contacting our corresponding author.

## Abbreviations

The following abbreviations are used in this manuscript:

ARA-C	Cytarabine
ASCT	Autologous stem cell transplantation
B2M	Beta-2 microglobulin
BCMA	B-cell maturation antigen
bsAb	Bispecific antibody
CNS	Central nervous system
CNS-MM	Central nervous system multiple myeloma
CSF	Cerebrospinal fluid
CT	Computed tomography
CyBorD	Cyclophosphamide, bortezomib, dexamethasone
Dara-Kd	Daratumumab, carfilzomib, dexamethasone
del17p	Deletion of chromosome 17p
DPd	Daratumumab, pomalidomide, dexamethasone
DRd	Daratumumab, lenalidomide, dexamethasone
DV-PACE	Dexamethasone, vincristine, cisplatin, doxorubicin, cyclophosphamide, etoposide
DXM	Dexamethasone
EMD	Extramedullary disease
FISH	Fluorescence in situ hybridisation
FLC	Free light chains
GPRC5D	G protein-coupled receptor class C group 5 member D
Ig	Immunoglobulin
ISS	International Staging System
IT	Intrathecal
KRD	Carfilzomib, lenalidomide, dexamethasone
LDH	Lactate dehydrogenase
Mel	Melphalan
MM	Multiple myeloma
MRI	Magnetic resonance imaging
MTX	Methotrexate
PAD	Bortezomib, doxorubicin, dexamethasone
PACE-Pd	Cisplatin, doxorubicin, cyclophosphamide, etoposide, pomalidomide, dexamethasone
PET-CT	Positron emission tomography–computed tomography
PomVD-PACE	Pomalidomide, bortezomib, dexamethasone, cisplatin, doxorubicin, cyclophosphamide, etoposide
RAD	Lenalidomide, doxorubicin, dexamethasone
RD	Lenalidomide, dexamethasone
R-ISS	Revised International Staging System
RT	Radiotherapy
SPEP	Serum protein electrophoresis
VGPR	Very good partial response
VRD	Bortezomib, lenalidomide, dexamethasone
WBLD-CT	Whole-body low-dose computed tomography
